# Kernel‐based active subspaces with application to computational fluid dynamics parametric problems using the discontinuous Galerkin method

**DOI:** 10.1002/nme.7099

**Published:** 2022-09-06

**Authors:** Francesco Romor, Marco Tezzele, Andrea Lario, Gianluigi Rozza

**Affiliations:** ^1^ Mathematics Area, mathLab, SISSA Scuola Internazionale Superiore di Studi Avanzati Trieste Italy

**Keywords:** active subspaces, dimension reduction, discontinuous Galerkin, kernel methods, ridge approximation

## Abstract

Nonlinear extensions to the active subspaces method have brought remarkable results for dimension reduction in the parameter space and response surface design. We further develop a kernel‐based nonlinear method. In particular, we introduce it in a broader mathematical framework that contemplates also the reduction in parameter space of multivariate objective functions. The implementation is thoroughly discussed and tested on more challenging benchmarks than the ones already present in the literature, for which dimension reduction with active subspaces produces already good results. Finally, we show a whole pipeline for the design of response surfaces with the new methodology in the context of a parametric computational fluid dynamics application solved with the discontinuous Galerkin method.

## 1 INTRODUCTION

Nowadays, in many industrial settings, the simulation of complex systems requires a huge amount of computational power. Problems involving high‐fidelity simulations are usually large‐scale, moreover the number of solutions required increases with the number of parameters. In this context, we mention optimization tasks, inverse problems, optimal control problems, and uncertainty quantification; they all suffer from the curse of dimensionality, that is, in this case, the computational time grows exponentially with the dimension of the input parameter space. Data‐driven reduced order methods (ROMs)^1^, ^2^, ^3^ have been developed to deal with such costly outer loop applications for parametric PDEs, but the limit for high dimensional parameter spaces remains.

One approach to alleviate the curse of dimensionality is to identify and exploit some notion of low‐dimensional structure of the model or objective function that maps the inputs to the outputs of interest. A possible linear input coordinate transformation technique is the sliced inverse regression (SIR)^4^ approach and its extensions.^5^, ^6^, ^7^ Sharing some characteristics with SIR, there is the active subspaces (AS) property*, ^8^, ^9^, ^10^, ^11^ which, in the last years, has emerged as a powerful linear data‐driven technique to construct ridge approximations using gradients of the model function. AS has been successfully applied to quantify uncertainty in the numerical simulation of the HyShot II scramjet,^12^ and for sensitivity analysis of an integrated hydrologic model.^13^ Reduction in parameter space has been coupled with model order reduction techniques^14^, ^15^, ^16^ to enable more complex numerical studies without increasing the computational load. We mention the use of AS in cardiovascular applications with POD‐Galerkin,^17^ in nonlinear structural analysis,^18^ in nautical and naval engineering,^19^, ^20^, ^21^, ^22^ coupled with POD with interpolation for structural and computational fluid dynamics (CFD) analysis,^23^, ^24^ and with dynamic mode decomposition in Reference 25. Applications in automotive engineering within a multi‐fidelity setting can be found in Reference 26, for turbomachinery, see Reference 27, while for results in chemistry, see References 28 and 29. Advances in efficient global design optimization with surrogate modeling are presented in References 30 and 31 and applied to the shape design of the N+2 Supersonic Passenger Jet. Applications to enhance optimization methods have been developed in References 32, 33, 34, 35. AS has also been successfully used to reduce the memory consumption of highly parameterized systems such as artificial neural networks.^36^, ^37^

Possible extensions and variants of the active subspaces property are the local active subspace method,^38^ the active manifold method^39^ which reduces the problem to the analysis of a 1D manifold by traversing the level sets of the model function at the expense of high online costs, the shared active subspace method,^40^ the active subspaces property for multivariate functions,^11^ and more recently an extension of AS to dynamical systems.^41^ Another method is nonlinear level set learning (NLL)^42^ which exploits RevNets to reduce the input parameter space with a nonlinear transformation.

The search for low dimensional structures is also investigated in machine learning with manifold learning algorithms. In this context, the active subspaces methodology can be seen as a supervised dimension reduction technique along with kernel principal component analysis (KPCA)^43^ and supervised kernel principal component analysis (SKPCA).^44^ Other methods in the context of kernel‐based ROMs are.^45^, ^46^, ^47^ In Reference 48, a nonlinear extension of the active subspaces property based on random Fourier features^49^, ^50^ is introduced and compared with machine learning manifold learning algorithms for the construction of Gaussian process regressions (GPR).^51^

From the preliminary work,^48^ in the context of supervised dimension reduction algorithms in machine learning, we develop the kernel‐based active subspaces (KAS) method. The novelties of our contribution are the following:

- Regarding the AS theoretical background, we provide an upper bound of the ridge approximation error (2) for vector‐valued objective functions and for a wide collection of probability distributions (see Assumption 3).
- We extend kernel‐based AS to vector‐valued model functions and develop a detailed algorithmic procedure for the optimization of the feature map. We also test different spectral measures (see Equation (20) for the definition), differently from Reference 48 where only the Gaussian measure is employed.
- The application to several test problems of increasing complexity. In particular, we mainly test KAS on problems where the active subspace is not present or the behavior is not linear, differently from Reference 48, where the comparison is made with KPCA and its variants on datasets with linear trends in the reduced parameter space, apart from the hyperparaboloid test case that we have also included among our toy problems.
- The KAS method is finally applied to a computational fluid dynamics problem and compared with the standard AS technique. We study the evolution of fluid flow past a NACA 0012 airfoil in a duct composed by an initialization channel and a chamber. The motion is modeled with the unsteady incompressible Navier–Stokes equations, and discretized with the discontinuous Galerkin (DG) method.^52^ Physical and geometrical parameters are introduced and sensitivity analysis of the lift and drag coefficients with respect to these parameters is provided.

The work is divided as follows: In Section 2, we briefly present the active subspaces property of a model function with a focus on the construction of Gaussian process response surfaces. Then, Section 3 illustrates the novel method called kernel‐based active subspaces for both scalar and vector‐valued model functions. Several tests to compare AS and KAS are provided in Section 4 where we start from scalar functions with radial symmetry, we analyze an epidemiology model and a vector‐valued output generated from a stochastic elliptic PDE. A parametric CFD test case for the study of the flow past a NACA airfoil using the DG method is presented in Section 5. Finally, we outline some perspectives and future studies in Section 6.

## 2 ACTIVE SUBSPACES FOR PARAMETER SPACE REDUCTION

Active subspaces (AS) approach proposed in Reference 8 and developed in Reference 9 is a technique for dimension reduction in parameter space. In brief, AS are defined as the leading eigenspaces of the second moment matrix of the model function's gradient (for scalar model functions) and constitutes a global sensitivity index.^11^ In the context of ridge approximation, the choice of the active subspace corresponds to the minimizer of an upper bound of the mean square error obtained through Poincaré‐type inequalities.^11^ After performing dimension reduction in the parameter space through AS, the method can be applied to reduce the computational costs of different parameter studies such as inverse problems, optimization tasks and numerical integration. In this work we are going to focus on the construction of response surfaces with Gaussian process regression.

Definition 1 (Hypothesis on input and output spaces) The quantities related to the input space are:

- m∈ℕ the dimension of the input space.
- (Ω,ℱ,P) the probability space.
- $X:(\Omega ,ℱ,P)\to {\mathbb{R}}^{m}$, the absolutely continuous random vector representing the parameters.
- $\rho :{\mathbb{R}}^{m}\to \mathbb{R}$, the probability density of X with support $𝒳\subset {\mathbb{R}}^{m}$.

The quantities related to the output are:

- d∈ℕ the dimension of the output space.
- $V=({\mathbb{R}}^{d},R_{V})$ the Euclidean space with metric $R_{V}\in ℳ(d\times d)$ ^†^ and norm
$$\|x{\|}_{R_{V}}^{2}=x^{T}R_{V}x.$$
- $f:𝒳\subset {\mathbb{R}}^{m}\to V$, the quantity/function of interest, also called objective function in optimization tasks.

Let $ℬ({\mathbb{R}}^{m})$ be the Borel σ‐algebra of ${\mathbb{R}}^{m}$. We will consider the Hilbert space $L^{2}({\mathbb{R}}^{m},ℬ({\mathbb{R}}^{m}),\rho \;;\;V)$, of the measurable functions $f:({\mathbb{R}}^{m},ℬ({\mathbb{R}}^{m}),\rho )\to ({\mathbb{R}}^{d},R_{V})$ such that

$$\|f{\|}_{L^{2}}^{2}:=\int_{𝒳}\|f(x){\|}_{R_{V}}^{2}\;d\rho (x)\leq \infty ;$$

and the Sobolev space $H^{1}({\mathbb{R}}^{m},ℬ({\mathbb{R}}^{m}),\rho \;;\;V)$ of measurable functions $f:({\mathbb{R}}^{m},ℬ({\mathbb{R}}^{m}),\rho )\to ({\mathbb{R}}^{d},R_{V})$ such that

(1) $$\|f{\|}_{H^{1}}^{2}:=\|f{\|}_{L^{2}}^{2}+\|\nabla f{\|}_{L^{2}}^{2}=\|f{\|}_{L^{2}}^{2}+|f{|}_{H^{1}}^{2}\leq \infty ,$$

where ∇f is the weak derivative of f, and $\|\nabla f{\|}_{L^{2}}=:|f{|}_{H^{1}}$.

We briefly recall how dimension reduction in parameter space is achieved in the construction of response surfaces. The first step involves the approximation of the model function with ridge approximation. We will follow References 11 and 53 for a review of the method.

The ridge approximation problem can be stated in the following way:

Definition 2 (Ridge approximation) Let $ℬ({\mathbb{R}}^{m})$ be the Borel σ‐algebra of ${\mathbb{R}}^{m}$. Given r∈ℕ,r≪d and a tolerance ϵ≥0, find the profile $h:({\mathbb{R}}^{m},ℬ({\mathbb{R}}^{m}),\rho )\to V$ and the r‐rank projection $P_{r}:{\mathbb{R}}^{m}\to {\mathbb{R}}^{m}$ such that

(2) $${𝔼}_{P}[\|f(X)-h(P_{r}X){\|}_{R_{V}}^{2}]\leq \epsilon^{2}.$$

In particular, we are interested in the minimization problem

(3) $$\underset{P_{r}\in ℳ(m\times m)}{\text{arg min}}\;{𝔼}_{P}\left[\|f(X)-\tilde{h}(P_{r}X){\|}_{R_{V}}^{2}\right],$$

where $\tilde{h}={𝔼}_{\rho }[f|\sigma (P_{r})]$ is the conditional expectation of f under the distribution ρ given the σ‐algebra $\sigma (P_{r})$. The range of the projector $P_{r}$, ${\mathbb{R}}^{r}∼\text{Im}(P_{r})\subset {\mathbb{R}}^{m}$, is the reduced parameter space. The kernel of the projector $P_{r}$, ${\mathbb{R}}^{m-r}∼\text{Im}(P_{r})\subset {\mathbb{R}}^{m}$, is the inactive subspace. The existence of $\tilde{h}$ is guaranteed by the Doob–Dynkin lemma.^54^ The function $\tilde{h}$ is proven to be the optimal profile for each fixed $P_{r}$, as a consequence of the definition of the conditional expectation of a random variable with respect to a σ‐algebra.

Dimension reduction is effective if the inequality (2) is satisfied for a specific tolerance. The choice of r is certainly of central importance. The dimension of the reduced parameter space can be chosen a priori for a specific parameter study (e.g., r‐dimensional regression), it can be chosen in order to satisfy the inequality (2) or it is determined to guarantee a good accuracy of the numerical method used to evaluate it.^55(Corollary3.10)^

Dividing the left term of the inequality (2) with ${𝔼}_{\rho }[\|f(X)-{𝔼}_{\rho }[f(X]){\|}_{R_{V}}^{2}]$ we obtain the Relative Root Mean Square Error (RRMSE) and since it is a normalized quantity, we will use it to make comparisons between different models

(4) $$\text{RRMSE}=\sqrt{\frac{{𝔼}_{P}[\|f(X)-h(P_{r}X){\|}_{R_{V}}^{2}]}{{𝔼}_{P}[\|f(X)-{𝔼}_{P}[f(X)]{\|}_{R_{V}}^{2}]}}.$$

We remark that $P_{r}$ is not unique. It can be shown that if $\tilde{h}$ is the optimal profile, then $P_{r}$ is not uniquely defined and can be chosen arbitrarily from the set $\left\{Q_{r}:{\mathbb{R}}^{m}\to {\mathbb{R}}^{m}|\;\ker Q_{r}=\ker P_{r}\right\}$, see Proposition 2.2 in Reference 11.

The following lemma is the key ingredient in the proof of the existence of an active subspace. It is inherently linked to probability Poincaré inequalities of the kind

(5) $$\int_{𝒳}\|h(x){\|}_{L^{2}}^{2}\;d\rho (x)\leq C_{P}(𝒳,\rho )\;\int_{𝒳}\|\nabla h(x){\|}_{L^{2}}^{2}\;d\rho (x),$$

for zero‐mean functions in the Sobolev space $h\in H^{1}(𝒳)$, where $C_{P}(𝒳,\rho )$ is the Poincaré constant dependent on the domain 𝒳 and on the probability density functions (p.d.f.), ρ. We need to make the following assumption to prove the next lemma and the next theorem.

Definition 3 The probability density function ρ:𝒳→ℝ belongs to one of the following classes:

1. 𝒳 is convex and bounded, $\exists \delta ,D>0:\;0<\delta \leq \|\rho (x){\|}_{L^{\infty }}\leq D<\infty \;\forall x\in 𝒳$.
2. ρ(x)∼exp(−V(x)) where $V:{\mathbb{R}}^{m}\to (-\infty ,\infty ]\;,V\in {𝒞}^{2}$ is α‐uniformly convex
(6) $$\begin{array}{ll} u^{T}\text{Hess}(V(x))u\geq \alpha \|u{\|}_{2}^{2},\;\forall x,u\in {\mathbb{R}}^{m}, & \end{array}$$
  where Hess(V(x)) is the Hessian of V(x).
3. ρ(x)∼exp(−V(x)) where V is a convex function. In this case, we require also f Lipschitz continuous.

In particular, the uniform distribution belongs to the first class, the multivariate Gaussian distribution 𝒩(m,∑) to the second with $\alpha =1/(\sigma_{\max}(\sum ))$ and the exponential and Laplace distributions to the third. A complete analysis of the various cases is done in Reference 53.

Proposition 1 *Let* (Ω,ℱ,P) *be a probability space,* $X:(\Omega ,ℱ,P)\to {\mathbb{R}}^{m}$ *an absolutely continuous random vector with probability density function* ρ *belonging to one of the classes from Assumption* *3* *. Then the following inequality is satisfied*

(7) $$\begin{array}{ll} {𝔼}_{\rho }\left[{\left(h-{𝔼}_{\rho }[h|\sigma (P_{r})]\right)}^{2}|\sigma (P_{r})\right]\leq C_{P}(P_{r},\rho )\;{𝔼}_{\rho }\left[\left\|(I-P_{r}^{T})\nabla h{\|}_{2}^{2}|\sigma (P_{r}\right)\right] & \end{array}$$

*for all scalar functions* $h\in H^{1}(𝒳)$ *and for all* r *‐rank orthogonal projectors,* $P_{r}$ *, where* $C_{P}(P_{r},\rho )$ *is the Poincaré constant depending on* $P_{r}$ *and on the p.d.f*. ρ.

A summary of the values of the Poincaré constant in relationship with the choice of the probability density function ρ is reported in Reference 53.

In the next theorem, the projection $P_{r}$ will depend on the output function f, so also the Poincaré constant $C_{P}(P_{r},\rho )$ will depend in fact on f.

We introduce the following notation for the matrix that substitutes the uncentered covariance matrix of the gradient ∇f in the case of the application of AS to scalar model functions^55^

$$H=\int_{𝒳}{\left(D_{x}f(x)\right)}^{T}R_{V}(\rho )(D_{x}f(x))\;d\rho (x),$$

where $D_{x}f(x)\in ℳ(d\times m)$ is the Jacobian matrix of f. The matrix $R_{V}(\rho )$ depends on the class which ρ belongs to, see the Appendix.

Theorem 1 (Existence of an active subspace) *Under Hypothesis* *1* *, let* $f\in H^{1}({\mathbb{R}}^{m},ℬ({\mathbb{R}}^{m}),\rho \;;\;V)$ *and let the p.d.f*. ρ *satisfy Lemma* *1* *and Assumption* *3* *. Then the solution* ${\tilde{P}}_{r}$ *of the ridge approximation problem* *2* *is the orthogonal projector to the eigenspace of the first* r *‐eigenvalues of* H *ordered by magnitude*

$$Hv_{i}=\lambda_{i}v_{i}\;\forall i\in \{1,\ldots ,m\},\;{\tilde{P}}_{r}=\overset{r}{\underset{j=1}{\sum }}v_{j}⊗v_{j},$$

*with* r∈ℕ *chosen such that*

(8) $${𝔼}_{\rho }\left[\|f-\tilde{h}{\|}_{R_{V}}^{2}\right]\leq C(C_{P},\tau )\;{\left(\overset{m}{\underset{i=r+1}{\sum }}\lambda_{i}\right)}^{\frac{1}{1+\tau }}\leq \epsilon^{2},$$

*with* $C(C_{P},\tau )$ *a constant depending on* τ>0 *related to the choice of* ρ *and on the Poincaré constant from lemma* *1* *, and* $\tilde{h}={𝔼}_{\rho }[f|\sigma (P_{r})]$ *is the conditional expectation of* f *given the* σ *‐algebra generated by the random variable* $P_{r}\circ X$.

This theorem summarizes the results from Propositions 2.5 and 2.6 of Reference 11, and from Lemmas 3.1, 4.2–4.4, and Theorem 4.5 of Reference 53. The proof is expanded in the Appendix.

The eigenspace $\text{span}\{v_{1},\ldots ,v_{r}\}\subset {\mathbb{R}}^{m}$ is the active subspace and the remaining eigenvectors generate the inactive subspace $\text{span}\{v_{r+1},\ldots ,v_{m}\}\subset {\mathbb{R}}^{m}$. The condition $f\in L^{2}({\mathbb{R}}^{m},ℬ({\mathbb{R}}^{m}),\rho \;;\;V)$ is necessary for f to satisfy the error bound (2).

For the explicit procedure to compute the active subspace given its dimension r, see Algorithm 1: from $W_{1}$ and $W_{2}$ we define the approximations of the projector $P_{r}$ with ${\hat{P}}_{r}=W_{1}W_{1}^{T}$.

Algorithm 1 **Active subspace computation**

1

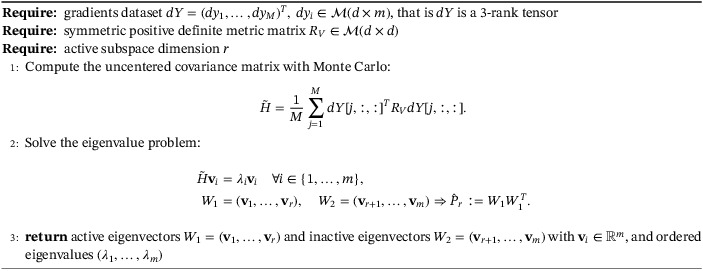

### 2.1 Response surfaces

The term response surface refers to the general procedure of finding the values of a model function f for new inputs without directly computing it but exploiting regression or interpolation from a training set $\left\{x_{i},\;f(x_{i})\right\}$. The procedure for constructing a Gaussian process response is reported in Algorithm 2, while in Algorithm 3, we show how to exploit it to predict the model function at new input parameters.

Directly applying the simple Monte Carlo method with N samples, we get a reduced approximation of f as

(9) $$({\tilde{h}}_{\epsilon }\circ P_{r})(X)={𝔼}_{\rho }\left[f|\sigma (P_{r})\right]\approx \frac{1}{N}\overset{N}{\underset{i=1}{\sum }}f({\hat{P}}_{r}X+(I_{d}-{\hat{P}}_{r})Y_{i})=:{ĥ}_{\epsilon ,N}({\hat{P}}_{r}X),$$

where we have made explicit the dependence of the optimal profile ${\tilde{h}}_{\epsilon }$ on ϵ, $Y_{1},\ldots ,Y_{N}$ are independent and identically distributed samples of Y∼ρ, and ${\hat{P}}_{r}$ is an approximation of $P_{r}$ obtained with the simple Monte Carlo method from H, see Algorithm 1. An intermediate approximation error is obtained employing the Poincaré inequality and the central limit theorem for the Monte Carlo approximation

(10) $${𝔼}_{P}\left[{\left(f(X)-{ĥ}_{\epsilon ,N}({\hat{P}}_{r}X)\right)}^{2}\right]\leq C_{1}{\left(1+N^{-1/2}\right)}^{2}(\lambda_{n+1}+\cdots +\lambda_{m}),$$

where $C_{1}$ is a constant, and $\lambda_{n+1},\ldots ,\lambda_{m}$ are the eigenvalues of the inactive subspace of H.^55^ (Theorem 4.4)

In practice, ${ĥ}_{\epsilon ,N}({\hat{P}}_{r}X)$ is approximated with a regression or an interpolation such that a response surface R satisfying ${𝔼}_{\rho }\left[\left({ĥ}_{\epsilon ,N}({\hat{P}}_{r}x)-R{\left({\hat{P}}_{r}x\right)}^{2}\right)\right]\leq C_{2}\delta$ is built, where $C_{2}$ is a constant, and δ depends on the chosen method. An estimate for the successive approximations

(11) $$f(X)\approx {\tilde{h}}_{\epsilon }(P_{r}X)\approx {ĥ}_{\epsilon ,N}({\hat{P}}_{r}X){\approx R}_{\epsilon ,N,\delta }({\hat{P}}_{r}X),$$

is given by

$$\begin{array}{ll} & {𝔼}_{P}\left[{\left(f(X)-R({\hat{P}}_{r}X)\right)}^{2}\right]\;\\ & \;\leq C_{1}{\left(1+N^{-1/2}\right)}^{2}{\left(\tau {\left(\lambda_{1}+\cdots +\lambda_{n}\right)}^{1/2}+{\left(\lambda_{n+1}+\cdots +\lambda_{m}\right)}^{1/2}\right)}^{2}+C_{2}\lambda ,\; \end{array}$$

where $\text{dist}(\text{Im}(P_{r}),\text{Im}({\hat{P}}_{r}))\leq \tau$, and $\lambda_{i}$ are the eigenvalues of H.^55^ (Theorem 4.8)

In our numerical simulations, we will build the response surface R with Gaussian process regression (GPR).^51^

Algorithm 2 **Response surface construction with Gaussian process regression over the active subspace**

1

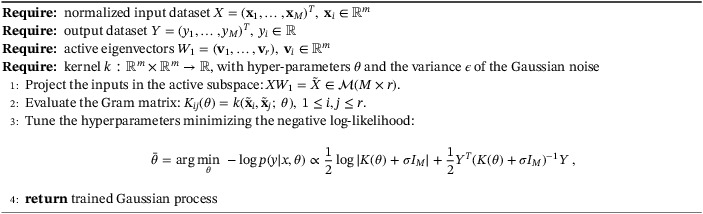

Algorithm 3 **Prediction phase using the Gaussian process response surface over the active subspace**

1

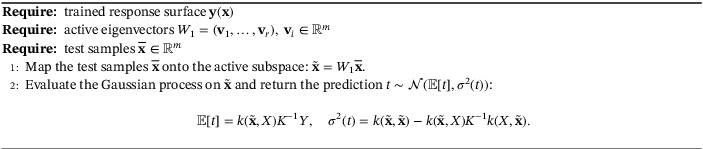

## 3 KERNEL‐BASED ACTIVE SUBSPACES EXTENSION

Keeping the notations of section 1, $X:(\Omega ,ℱ,P)\to {\mathbb{R}}^{m}$ is the absolutely continuous random vector representing the m‐dimensional inputs with density $\rho :𝒳\subset {\mathbb{R}}^{m}\to \mathbb{R}$, and $f:𝒳\subset {\mathbb{R}}^{m}\to (V,R_{V})$ is the model function that we assume to be continuously differentiable and Lipschitz continuous.

One drawback of sufficient dimension reduction with AS applied to ridge approximation is that if a clear linear trend is missing, projecting the inputs as $P_{r}X$ represents a loss of accuracy on the approximation of the model f that may not be compensated even by the choice of the optimal profile $\tilde{h}\circ P_{r}={𝔼}_{\rho }[f|\sigma (P_{r})]$. In order to overcome this, nonlinear dimension reduction to one‐dimensional parameter space could be achieved discovering a curve in the space of parameters that cuts transversely the level sets of f, this variation is presented in Reference 39 as active manifold. Another approach could consist in finding a diffeomorphism ϕ that reshapes the level sets such that subsequently applying AS dimension reduction to the new model function $\tilde{f}\circ \phi =f$ could be more profitable:


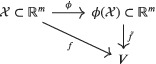


Unfortunately constructing the active manifold or finding the right diffeomorphism ϕ could be a complicated matter. If we renounce to have a backward map and we weaken the bond of the method with the model, we can consider an immersion ϕ from the space of parameters 𝒳 to an infinite‐dimensional Hilbert space ℍ obtaining


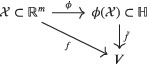


**FIGURE 1:**
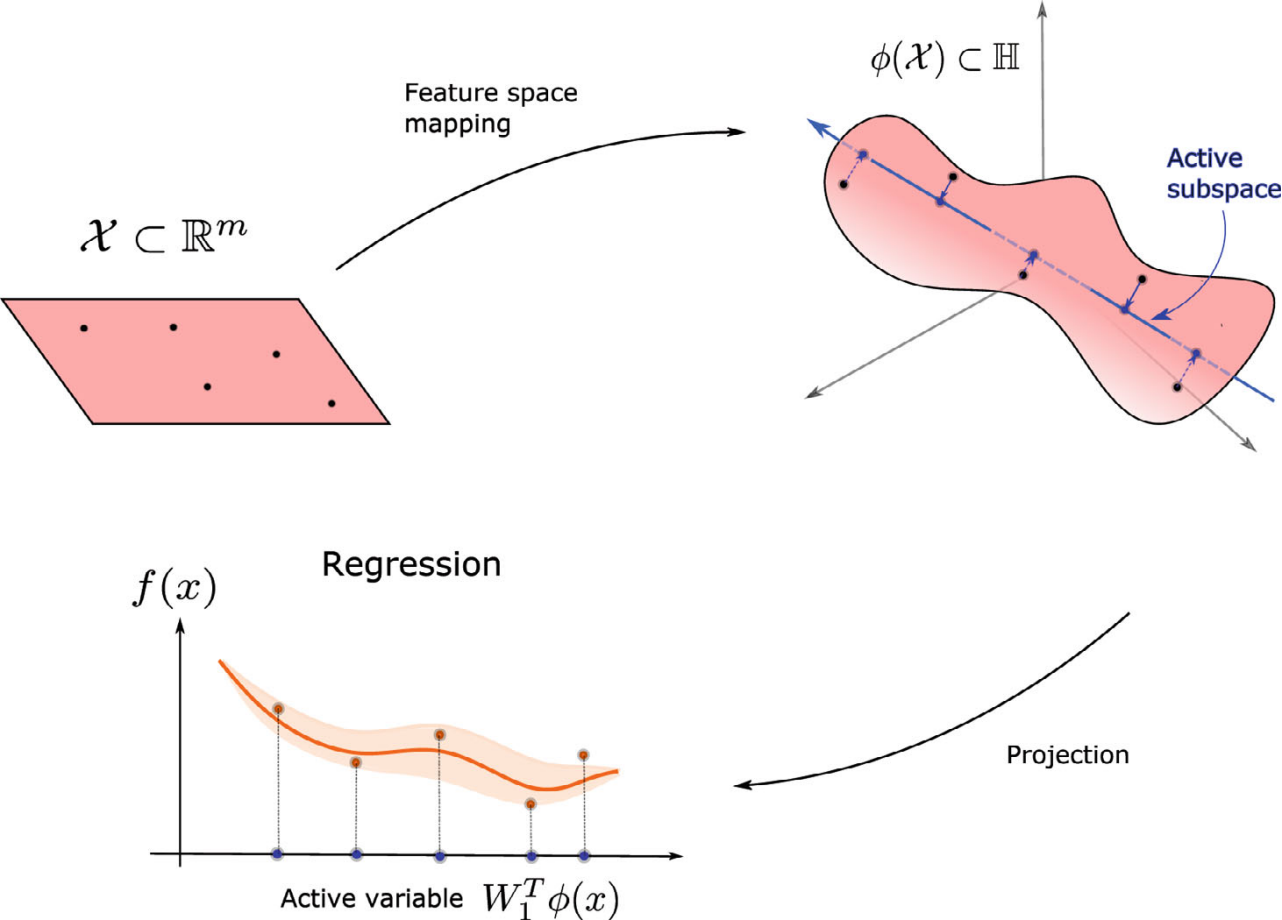
Illustration of the construction of a one‐dimensional response surface with kernel‐based active subspaces and Gaussian process regression

This is a common procedure in machine learning in order to increase the number of features.^51^ Then AS is applied to the new model function $\tilde{f}:\phi (𝒳)\subset ℍ\to V$ with parameter space ϕ(𝒳)⊂ℍ. A response surface can be built with Algorithm 2 remembering to replace every occurrence of the inputs x with their images ϕ(x). A synthetic scheme of the procedure is represented in Figure 1.

In practice, we consider a discretization of the infinite‐dimensional Hilbert space ${\mathbb{R}}^{D}≃ℍ$ with D>m. Dimension reduction with AS results in the choice of a r‐rank projection in the much broader set of r‐rank projections in ℍ.

Since for AS only the samples of the Jacobian matrix of the model function are employed, we can ignore the definition of the new map $\tilde{f}:\phi (𝒳)\subset ℍ\to (V,R_{V})$ and focus only on the computation of the Jacobian matrix of $\tilde{f}$ with respect to the new input variable z:=ϕ(x). The uncentered covariance matrix becomes

$$\begin{array}{ll} H & =\int_{\phi (𝒳)}\left[{\left(D_{z}\tilde{f}\right)}^{T}(z)\right]R_{V}\left[\left(D_{z}\tilde{f})(z\right)\right]d\mu (z)\;\\ & =\int_{𝒳}\left[{\left(D_{z}\tilde{f}\right)}^{T}(\phi (x))\right]R_{V}\left[\left(D_{z}\tilde{f})(\phi (x)\right)\right]d{ℒ}_{X}(x),\; \end{array}$$

where $\mu :=\phi_{\#}({ℒ}_{X})$ is the pushforward probability measure of ${ℒ}_{X}$ (the law of probability of X) with respect to the map ϕ. Simple Monte Carlo can be applied sampling from the distribution ρ in the input space 𝒳

$$\begin{array}{ll} H & =\int_{𝒳}\left[{\left(D_{z}\tilde{f}\right)}^{T}(\phi (x))\right]R_{V}\left[\left(D_{z}\tilde{f})(\phi (x)\right)\right]d{ℒ}_{X}(x)\;\\ & \approx \frac{1}{M}\overset{M}{\underset{i=1}{\sum }}\left[{\left(D_{z}\tilde{f}\right)}^{T}(\phi (x_{i}))\right]R_{V}\left[\left(D_{z}\tilde{f})(\phi (x_{i})\right)\right].\; \end{array}$$

The gradients of $\tilde{f}$ with respect to the new input variable Z are computed from the known values $D_{x}f$ with the chain rule.

The application of the chain rule to the composition of functions $\tilde{f}\circ \phi :{\mathbb{R}}^{m}\to ℍ\to V$ is applicable if $\tilde{f}$ is defined in an open set U⊃ϕ(𝒳). If ϕ is nonsingular and also injective the new input space is a m‐dimensional submanifold of ℍ. If ϕ is also smooth there exists a smooth extension of $\tilde{f}:\phi (𝒳)\subset ℍ\to V$ onto the whole domain ℍ, see Proposition 1.36 from Reference 56.

If the Hilbert space ℍ has finite dimension $ℍ∼{\mathbb{R}}^{D}$ this procedure leaves us with an underdetermined linear system to solve for $D_{z}\tilde{f}$

(12) $$\begin{array}{ll} & D_{z}\tilde{f}(\phi (x))D\phi (x)=D_{x}f(x),\\ & D_{z}\tilde{f}(\phi (x))=D_{x}f(x){\left(D\phi (x)\right)}^{†}, \end{array}$$

where ${}^{†}$ stands for the right Moore–Penrose inverse of the matrix Dϕ(x) with rank r, that is

$${\left(D\phi (x)\right)}^{†}=V\sum^{†}U^{T},$$

with the usual notation for the singular value decomposition (SVD) of Dϕ(x)

(13) $$D\phi (x)=U\sum V^{T},$$

and $\sum^{†}\in ℳ(r\times r)$ equal to the diagonal matrix with the inverse of the singular values as diagonal elements. As anticipated if f is smooth enough and ϕ is an embedding, so that Dϕ has full rank, the previous system has an unique solution. The most crucial part is the evaluation of the gradients $D_{x}f(x)$ from the input output couples, when they are not available analytically or from the adjoint method applied to PDEs models: different approaches are present in the literature, like local polynomial regressions and Gaussian process regression on the whole domain to approximate the gradients; both are available in the ATHENA package.^57^ For an estimate of the ridge approximation error due to inaccurate gradients, see Reference 9.

Finally, we remark that in the AS method we approximate the random variable X as

(14) $$P_{r}X=v_{1}(v_{1}\cdot X)+\cdots +v_{r}(v_{r}\cdot X),$$

with $\{v_{i}\}\subset {\mathbb{R}}^{m}$ the active eigenvectors, whereas with KAS the reduced input space is contained in ℋ

(15) $$P_{r}X=v_{1}(v_{1}\cdot \phi (X))+\cdots +v_{r}(v_{r}\cdot \phi (X)),$$

with $\{v_{i}\}\subset ℋ$ the active eigenvectors of KAS. In this case, the model is enriched by the nonlinear feature map ϕ.

Algorithm 4 **Kernel‐based active subspace computation**

1

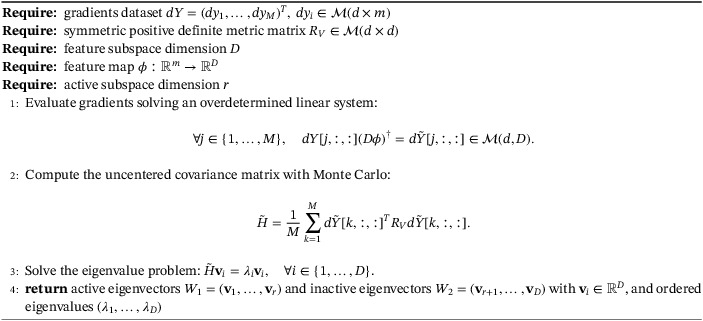

### 3.1 Choice of the feature map

The choice for the map ϕ is linked to the theory of reproducing kernel Hilbert spaces (RKHS),^58^ and it is defined as

(16) $$\begin{array}{ll} z=\phi (x) & =\sqrt{\frac{2}{D}}\;\sigma_{f}\;\cos(Wx+b), \end{array}$$

(17) $$\begin{array}{ll} \cos(Wx+b) & :=\frac{1}{\sqrt{D}}{\left(\cos(W[1,:]\cdot x+b_{1}),\ldots ,\cos(W[D,:]\cdot x+b_{D})\right)}^{T}, \end{array}$$

where $\sigma_{f}$ is an hyperparameter corresponding to the empirical variance of the model, W∈ℳ(D×m) is the projection matrix whose rows are sampled from a probability distribution μ on ${\mathbb{R}}^{m}$ and $b\in {\mathbb{R}}^{D}$ is a bias term whose components are sampled independently and uniformly in the interval [0,2π]. We remark that its Jacobian can be computed analytically as

(18) $$\frac{\partial z^{j}}{\partial x^{i}}=-\sqrt{\frac{2}{D}}\;\sigma_{f}\;\sin\left(\overset{D}{\underset{k=1}{\sum }}W_{ik}x_{k}+b_{k}\right)\;W_{ij},$$

for all i∈{1,…,m}, and for all j∈{1,…,D}.

We remark that in order to guarantee the correctness of the procedure for evaluating the gradients we have to prove that the feature map is injective and nonsingular. In general, however, the feature map (16) cannot not be injective due to the periodicity of the cosine but at least it is almost surely nonsingular if the dimension of the feature space is high enough.

The feature map (16) is not the only effective immersion that provides a kernel‐based extension of the active subspaces. For example an alternative is the following composition of a linear map with a sigmoid

$$\phi (z)=\frac{C}{1+\alpha \;e^{-Wz}},$$

where C is a constant, α is an hyperparameter to be tuned, and W∈ℳ(D,m) is, as before, a matrix whose rows are sampled from a probability distribution on ${\mathbb{R}}^{m}$.

Other choices involve the use of deep neural networks to learn the profile h and the projection function $P_{r}$ of the ridge approximation problem.^59^

The tuning of the hyperparameters of the spectral measure consists in a global optimization problem where the dimension of the domain can vary between 1 and the dimension of the input space m. The object function to optimize is the relative root mean square error (RRMSE)

(19) $$\text{RRMSE}(Y_{\text{test}},T_{\text{test}})=\sqrt{\frac{\overset{N}{\underset{i=1}{\sum }}{\left(t_{i}-y_{i}\right)}^{2}}{\overset{N}{\underset{i=1}{\sum }}{\left(t_{i}-\bar{y}\right)}^{2}}},$$

where $T_{\text{test}}={\left(t_{i}\right)}_{i\in \{1,\ldots ,N\}}$ are the predictions obtained from the response surface built with KAS and associated to the test set, $Y_{\text{test}}={\left(y_{i}\right)}_{i\in \{1,\ldots ,N\}}$ are the targets associated to the test set, and $\bar{y}$ is the mean value of the targets. We implemented a logarithmic grid‐search, see Algorithm 5, making use of the SciPy library.^60^ Another choice could be Bayesian stochastic optimization implemented in the open‐source library GPyOpt.^61^

The tuning of the hyperparameters of the spectral measure chosen is the most computationally expensive part of the procedure. We report the computational complexity of the algorithms introduced to have a better understanding of the additional cost implied by the implementation of response surface design with KAS. Let us assume that the number of random Fourier features D, the number of input, output, and gradient samples M, and the dimension of the parameter space m, are ordered in this manner D>M>m, as is usually the case, and that the quantity of interest f is a scalar function. The cost of computing an active subspace is $O(Mm^{2})$, that is the cost of the SVD of the gradients matrix dY used to get the active and inactive eigenvectors in Algorithm 1. The cost of the training of a response surface with Gaussian process regression in Algorithm 2 depends on the cost of minimization of the log‐likelihood: each evaluation of the log‐likelihood involves the computation of the determinant and the inverse of the regularized Gram matrix $K(\theta )+\sigma I_{M}$, that is $O(M^{3})$. Finally, the cost for the evaluation of the kernel‐based active subspace is associated to the SVD of $d\tilde{Y}$ that is $O(DM^{2})$ in Algorithm 4, and to the resolution of the overdetermined linear system to obtain the gradients $d\tilde{Y}$, that is M times $O(Dm^{2})$ since it is related to the evaluation of the pseudo‐inverse of Dϕ. So, the computational complexity for the response surface design with AS and GPR is $O(n_{\text{GPR}}M^{3})$, while for the response surface design with KAS and GPR is $O\left(n_{\text{grid‐search}}n\left(D\frac{M^{2}}{n^{2}}+\frac{M}{n}Dm^{2}+n_{\text{GPR}}\frac{M^{3}}{n^{3}}\right)\right)$, where $n_{\text{GPR}}$ is the maximum number of steps of the optimization algorithm used to minimize the log‐likelihood, $n_{\text{grid‐search}}$ is the number of hyperparameter instances γ∈G to try in Algorithm 5, and n is the number of batches in the n‐fold cross validation procedure. In particular, for each grid search hyperparameter the main cost is associated to the GPR training since $n_{\text{GPR}}$ usually satisfy $Dn<n_{\text{GPR}}M$, when the optimizer chosen is L‐BFGS‐B from SciPy,^60^ accounting also for the number of restarts of the optimizer: in the numerical tests we performed the number of restarts of the training of the GPR is problem‐dependent but always less than 10. In general, the number $n_{\text{grid‐search}}$ depends on the chosen application, and the multiplicative factor between the computational complexity of the response surface design procedure with KAS or AS is lower than $3n_{\text{grid‐search}}n$.

Algorithm 5 **Tuning the feature map with logarithmic grid‐search**

1

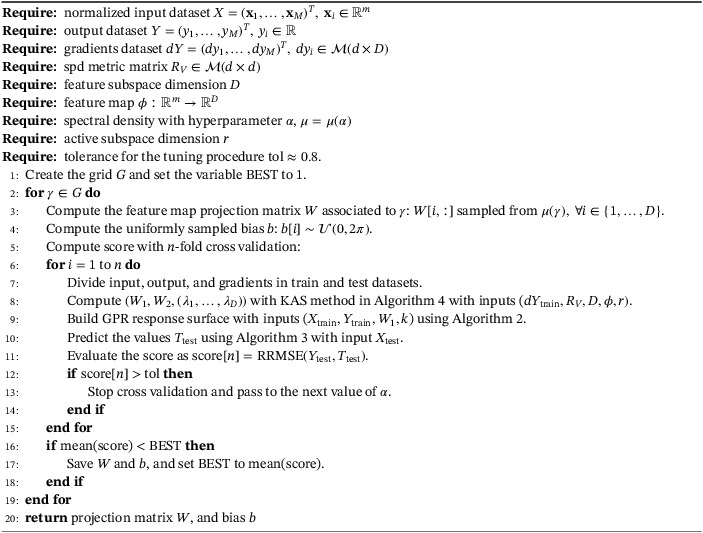

### 3.2 Random Fourier features

The motivation behind the choice for this map from Equation (16) comes from the theory on RKHS. The infinite‐dimensional Hilbert space (ℍ,⟨·,·⟩) is assumed to be a RKHS with real shift‐invariant kernel k:𝒳×𝒳→ℝ with k(0)=1 and feature map ϕ.

In order to get a discrete approximation of $\phi :𝒳\subset {\mathbb{R}}^{m}\to ℍ$, random Fourier features are employed.^49^, ^50^ Bochner's theorem^62^ guarantees the existence of a spectral probability measure μ such that

(20) $$k(x,y)=\int_{{\mathbb{R}}^{m}}e^{i\omega \cdot (x-y)}\;d\mu (\omega ).$$

From this identity, we can get a discrete approximation of the scalar product ⟨·,·⟩ with Monte Carlo method, exploiting the fact that the kernel is real

(21) $$\begin{array}{ll} \left\langle \phi (x),\phi (y)\right\rangle & =k(x,y)\approx \frac{1}{D}\overset{D}{\underset{i=1}{\sum }}\cos(\omega_{i}\cdot x+b_{i})\cos(\omega_{i}\cdot y+b_{i})=z^{T}z, \end{array}$$

(22) $$\begin{array}{ll} z & =\frac{1}{\sqrt{D}}(\cos(\omega_{1}\cdot x+b_{1}),\ldots ,\cos(\omega_{D}\cdot x+b_{D})), \end{array}$$

and from this relation we obtain the approximation ϕ≈z. The sampled vectors ${\left\{\omega_{i}\right\}}_{i=1,\ldots ,D}$ are called random Fourier features. The scalars $\{b_{i}\}{}_{i=1,\ldots ,D}$ are bias terms introduced since in the approximation we have excluded some trigonometric terms from the following initial expression

$$\frac{1}{D}\overset{D}{\underset{i=1}{\sum }}\left(\cos(\omega_{i}\cdot x)\cos(\omega_{i}\cdot y)-\sin(\omega_{i}\cdot x)\sin(\omega_{i}\cdot y)\right).$$

Random Fourier features are frequently used to approximate kernels. We consider only spectral probability measures which have a probability density, usually named spectral density. In the approximation of the kernel with random Fourier features, under some regularity conditions on the kernel, an explicit probabilistic bound depending on the dimension of the feature space D can be proved.^62^ This technique is used to scale up kernel principal component analysis^63^, ^64^ and supervised kernel principal component analysis,^44^ but in the case of kernel‐based AS the resulting overdetermined linear system employed to compute the Jacobian matrix of the new model function increases in dimension instead.

The most famous kernel is the squared exponential kernel also called Radial Basis Function kernel (RBF)

(23) $$k_{\text{RBF}}(x,y)=\exp\left(-\frac{\|x-y{\|}^{2}}{2l^{2}}\right),$$

where l is the characteristic length‐scale. The spectral density is Gaussian $𝒩(0,\;1/4\pi^{2}l^{2})$:

(24) $$S(\omega )={\left(2\pi l^{2}\right)}^{D/2}\exp(-2\pi^{2}l^{2}\omega^{2}).$$

Thanks to Bochner's theorem to every probability distribution that admits a probability density function corresponds a stationary positive definite kernel. So having in mind the definition of the feature map ϕ from Equation (16), we can choose any probability distribution for sampling the random projection matrix W∈ℳ(D×m) without focusing on the corresponding kernel since it is not needed by the numerical procedure.

After the choice of the spectral measure the corresponding hyperparameters have to be tuned. This is linked to the choice of the hypothesis models in machine learning and it is usually carried out for the hyperparameters of the employed kernel. From the choice of the kernel and the corresponding hyperparameters, some regularity properties of the model are implicitly assumed.^51^

## 4 BENCHMARK TEST PROBLEMS

In this section, we are going to present some benchmarks to prove the potential gain of KAS over standard linear AS, for both scalar and vectorial model functions. In particular, we test KAS on radial symmetric functions, with 2‐dimensional and 8‐dimensional parameter spaces, on the approximation of the reproduction number $R_{0}$ of the SEIR model, and finally on a vectorial output function that is the solution of a Poisson problem.

One‐dimensional response surfaces are built following the algorithm described in Section 2.1. The tuning of the hyperparameters of the feature map is carried out with a logarithmic grid‐search and 5‐fold cross validation for the Ebola test case, while for the other cases we employed Bayesian stochastic optimization implemented in Reference 65 with 3‐fold cross validation. The score function chosen is the relative root mean square error (RRMSE). The spectral measure for each test case is chosen by brute force among the Laplace, Gaussian, Beta, and multivariate Gaussian distributions. The number of Fourier features is not established based on a criterion but we have seen experimentally that above a certain threshold the number of features is high enough to at least reproduce the accuracy of the AS method. Since the most sensitive part to the final accuracy of the response surface is the tuning of the hyperparameters of the spectral measures, we suggest to choose an affordable number of features between 1000 and 2000, and focus on the tuning of said hyperparameters instead.We remark that the number of samples employed is problem dependent: some heuristics to determine it can be found in Reference 9, but the crucial point is that additional training samples with respect to the ones used for the AS method are not needed for the novel KAS method. Moreover, the CPU time for the hyperparameters tuning procedure is usually negligible with respect to the time required to obtain input‐output pairs from the numerical simulation of PDEs models: in our applications the tuning procedure's computational time is in the order of minutes (usually around 10–15 min for most testcases), while for the CFD application of Section 5 it is in the order of days and for the stochastic elliptic partial differential equation of Section 4.3 it is in the order of hours. We also remark that the tuning Algorithm 5, the GPR training restarts, and the choice of the spectral measure can be easily parallelized.

For the radial symmetric and Ebola test cases, the inputs are sampled from a uniform distribution with problem dependent ranges. For the stochastic elliptic partial differential case, the inputs are the coefficients of a Karhunen–Loève expansion and are sampled from a normal distribution. All the computations regarding AS and KAS are done using the open source Python package called ATHENA.^57^

### 4.1 Radial symmetric functions

Radial symmetric functions represent a class of model functions for which AS is not able to unveil any low dimensional behavior. In fact for these functions any rotation of the parameter space produce the same model representation. Instead kernel‐based AS is able to overcome this problem thanks to the mapping onto the feature space.

We present two benchmarks: an 8‐dimensional hyperparaboloid defined as

(25) $$f:{\left[-1,1\right]}^{8}\subset {\mathbb{R}}^{8}\to \mathbb{R},\;f(x)=\frac{1}{2}\|x{\|}^{2},$$

and the surface of revolution in ${\mathbb{R}}^{3}$ with generatrix $g(x)=\sin(x^{2})$

(26) $$f:{\left[-3,3\right]}^{2}\subset {\mathbb{R}}^{2}\to \mathbb{R},\;f(x)=g(\|x\|)=\sin(\|x{\|}^{2}).$$

The gradients are computed analytically.

For the hyperparaboloid we use $N_{s}=500$ independent, uniformly distributed training samples in ${\left[-1,1\right]}^{8}$, while for the sine case the training samples are $N_{s}=800$ in ${\left[-3,3\right]}^{2}$. In both cases, the test samples are 500. The feature space has dimension 1000 for both the first and the second case. The spectral distribution chosen is the multivariate normal with hyperparameter a uniform variance $\lambda I_{d}$, and a product of Laplace distributions with γ and b as hyperparameters, respectively. The tuning is carried out with 3‐fold cross validation. The results are summarized in Table 1.

**TABLE 1:** Performance results for AS and KAS methods

Case	Dim	$N_{s}$	Spectral distribution	Feature space dim	RRMSE AS	RRMSE KAS
Hyperparaboloid	8	500	$𝒩(0,\lambda I_{d})$	1000	0.98 ± 0.03	**0.23** ± 0.02
Sine	2	800	Laplace(γ,b)	1000	1.011 ± 0.01	**0.31** ± 0.06
Ebola	8	800	Beta(α,β)	1000	0.46 ± 0.31	**0.31** ± 0.03
SPDE (31)	10	1000	𝒩(0,∑)	1500	0.611 ± 0.001	**0.515** ± 0.013

*Note*: For each case, we report the parameter space dimension, the number of samples $N_{s}$ used for the training, the chosen distribution, the dimension of the feature space, and the RRMSE mean and standard deviation for AS and KAS. The best results are given in bold.

**FIGURE 2:**
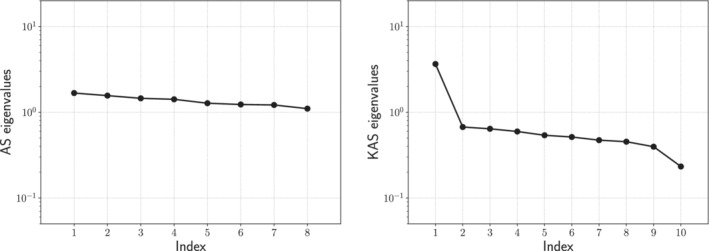
Eigenvalues of the covariance matrix $\tilde{H}\in {\mathbb{R}}^{8\times 8}$ applied to the hyperparaboloid case for the AS procedure on the left, and the first 10 eigenvalues of the covariance matrix $\tilde{H}\in {\mathbb{R}}^{1000\times 1000}$ for the KAS procedure applied to the same case on the right

Looking at the eigenvalues of the uncentered covariance matrix of the gradients $\tilde{H}$ for the hyperparaboloid case in Figure 2, we can clearly see how the decay for AS is almost absent, while using KAS the decay after the first eigenvalue is pronounced, suggesting the presence of a kernel‐based active subspace of dimension 1.

**FIGURE 3:**
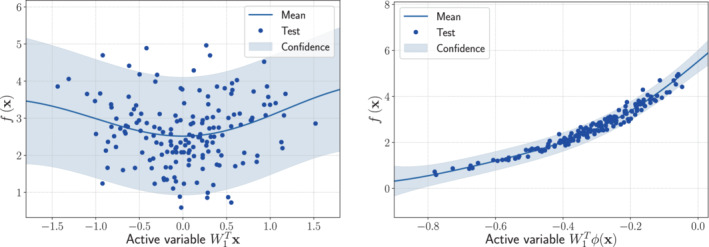
Comparison between the sufficiency summary plots obtained from the application of AS and KAS methods for the hyperparaboloid model function with domain ${\left[-1,1\right]}^{8}$, defined in Equation (25). The left plot refers to AS, the right plot to KAS. With the blue solid line, we depict the posterior mean of the GP, with the shadow area the 68% confidence intervals, and with the blue dots the testing points.

**FIGURE 4:**
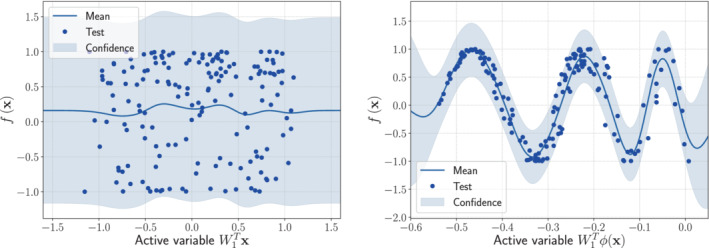
Comparison between the sufficiency summary plots obtained from the application of AS and KAS methods for the surface of revolution model function with domain ${\left[-3,3\right]}^{2}$, defined in Equation (26). The left plot refers to AS, the right plot to KAS. With the blue solid line, we depict the posterior mean of the GP, with the shadow area the 68% confidence intervals, and with the blue dots the testing points.

The one‐dimensional sufficient summary plots, which are f(x) against $W_{1}^{T}x$—in the AS case—or against $W_{1}^{T}\phi (x)$—in the KAS case, are shown in Figures 3 and 4, respectively. On the left panels, we present the Gaussian process response surfaces obtained from the active subspaces reduction, while on the right panels the ones obtained with the kernel‐based AS extension. As we can see AS fails to properly reduce the parameter spaces, since there are no preferred directions over which the model functions vary the most. The KAS approach, on the contrary, is able to unveil the corresponding generatrices. This results in a reduction of the RMS by a factor of at least 3 (see Table 1).

### 4.2 SEIR model for the spread of Ebola

In most engineering applications, the output of interest presents a monotonic behavior with respect to the parameters. This means that, for example, the increment in the inputs produces a proportional response in the outputs. Rarely, the model function has a radial symmetry, and in such cases the parameter space can be divided in subdomains, which are analyzed separately. In this section, we are going to present a test case where there is no radial symmetry, showing that, even in this case the kernel‐based AS presents better performance with respect to AS.

For the Ebola test case,^‡^ the output of interest is the basic reproduction number $R_{0}$ of the SEIR model, described in Reference 66, which reads

(27) $$R_{0}=\frac{\beta_{1}+\frac{\beta_{2}\rho_{1}\gamma_{1}}{\omega }+\frac{\beta_{3}}{\gamma_{2}}\psi }{\gamma_{1}+\psi },$$

with parameters distributed uniformly in $\Omega \subset {\mathbb{R}}^{8}$. The parameter space Ω is an hypercube defined by the lower and upper bounds summarized in Table 2.

**TABLE 2:** Parameter ranges for the Ebola model

	$\beta_{1}$	$\beta_{2}$	$\beta_{3}$	$\rho_{1}$	$\gamma_{1}$	$\gamma_{2}$	ω	ψ
Lower bound	0.1	0.1	0.05	0.41	0.0276	0.081	0.25	0.0833
Upper bound	0.4	0.4	0.2	1	0.1702	0.21	0.5	0.7

*Note*: Data taken from Reference 66.

**FIGURE 5:**
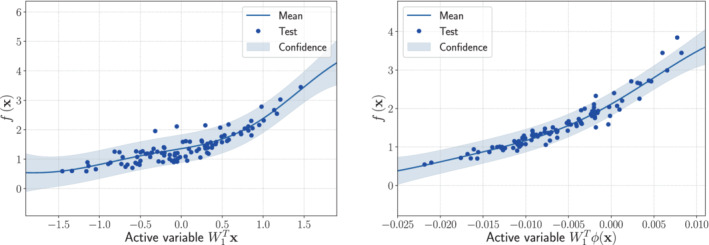
Comparison between the sufficiency summary plots obtained from the application of AS and KAS methods for the $R_{0}$ model function with domain Ω, defined in Equation (27). The left plot refers to AS, the right plot to KAS. With the blue solid line, we depict the posterior mean of the GP, with the shadow area the 68% confidence intervals, and with the blue dots the testing points.

We can compare the two one‐dimensional response surfaces obtained with Gaussian process regression. The training samples are $N_{s}=800$, and we use 1000 features. As spectral measure we use again the multivariate Gaussian distribution 𝒩(0,∑) with hyperparameters the elements of the diagonal of the covariance matrix. The tuning is carried out with 5‐fold cross validation. Even in this case, the KAS approach results in smaller RMS with respect to the use of AS (around 60% less), as reported in Table 1. In Figure 5, we report the comparison of the two approaches over an active subspace of dimension 1.

### 4.3 Elliptic partial differential equation with random coefficients

In our last benchmark, we apply the kernel‐based AS to a vectorial model function, that is the solution of a Poisson problem with heterogeneous diffusion coefficient. We refer to Reference 11 for an application, on the same problem, of the AS approach.

We consider the following stochastic Poisson problem on the square $x=(x,y)\in \Omega :={\left[0,1\right]}^{2}$:

(28) $$\left\{\begin{array}{ll} -\nabla \cdot (\kappa \;\nabla u)=1,\; & x\in \Omega ,\\ u=0,\; & x\in \partial \Omega_{\text{top}}\cup \partial \Omega_{\text{bottom}},\\ u=10y(1-y),\; & x\in \partial \Omega_{\text{left}},\\ n\cdot \nabla u=0,\; & x\in \partial \Omega_{\text{right}}, \end{array}\right.$$

with homogeneous Neumann boundary condition on the right side of the domain, that is $\partial \Omega_{\text{right}}$, Neumann boundary conditions on the left side of the domain, that is $\partial \Omega_{\text{left}}$, and Dirichlet boundary conditions on the remaining part of ∂Ω. The diffusion coefficient κ:(Ω,𝒜,P)×Ω→ℝ, where 𝒜 is a σ‐algebra, is such that log(κ) is a Gaussian random field, with covariance function C(x,y) defined by

(29) $$C(x,y)=\exp\left(-\frac{\|x-y{\|}^{2}}{\beta^{2}}\right),\;\forall x,y\in \Omega ,$$

where β=0.03 is the correlation length. This random field is approximated with the truncated Karhunen–Loève decomposition

(30) $$\kappa (s,x)\approx \exp\left(\overset{m}{\underset{i=0}{\sum }}X_{i}(s)\gamma_{i}\psi_{i}(x)\right),\;\forall (s,x)\in \Omega \times \Omega ,$$

where ${\left(X_{i}\right)}_{i\in 1,\ldots ,m}$ are independent standard normal distributed random variables, and ${\left(\gamma_{i},\psi_{i}\right)}_{i\in 1,\ldots ,d}$ are the eigenpairs of the Karhunen–Loève decomposition of the zero‐mean random field κ.

In our simulation, the domain Ω is discretized with a triangular unstructured mesh 𝒯 with 3194 triangles. The parameter space has dimension m=10. The simulations are carried out with the finite element method (FEM) with polynomial order one, and for each simulation the parameters ${\left(X_{i}\right)}_{i=1,\ldots ,m}$ are sampled from a standard normal distribution. The solution u is evaluated at d=1668 degrees of freedom, thus $\left(V,R_{V})\approx ({\mathbb{R}}^{d},S+M\right)$ where the metric $R_{V}$ is approximated with the sum of the stiffness matrix $S\in {\mathbb{R}}^{d}\times {\mathbb{R}}^{d}$ and the mass matrix $M\in {\mathbb{R}}^{d}\times {\mathbb{R}}^{d}$. This sum is a discretization of the norm of the Sobolev space $H^{1}(\Omega )$. The number of features used in the KAS procedure is D=1500, the number of different independent simulations is M=1000.

**FIGURE 6:**
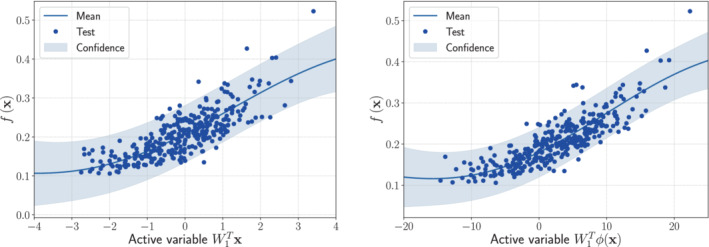
Comparison between the sufficiency summary plots obtained from the application of AS and KAS methods for the stochastic PDE model, defined in Equations (28) and (31). The left plot refers to AS, the right plot to KAS. With the blue solid line, we depict the posterior mean of the GP, with the shadow area the 68% confidence intervals, and with the blue dots the testing points.

Three outputs of interest are considered. The first target function $f:{\mathbb{R}}^{m}\to \mathbb{R}$ is the mean value of the solution at the right boundary $\partial \Omega_{\text{right}}$, which reads

(31) $$f(X)=\frac{1}{\left|\partial \Omega_{\text{right}}\right|}\int_{\partial \Omega_{\text{right}}}u(s)\;ds,$$

and it is used to tune the feature map minimizing the RRMSE of the Gaussian process regression, as described in Algorithm 5. A summary of the results for the first output is reported in Table 1. The plots of the regression are reported in Figure 6. Even in this case both from a qualitative and a quantitative point of view, the kernel‐based approach achieves the best results.

The second output we consider is the solution function

(32) $$f:{\mathbb{R}}^{m}\to (V,R_{V})\approx ({\mathbb{R}}^{d},S),\;f(X)=u\in {\mathbb{R}}^{d}.$$

This output can be employed as a surrogate model to predict the solution u given the parameters X that define the diffusion coefficient instead of carrying out the numerical simulation. The surrogate model should be constructed over the span of the modes identified by the chosen reduction strategy, after projecting the data. AS and KAS modes are distinguished but can detect some common regions of interest as shown in Table 3.

**TABLE 3:** First 3 modes using Karhunen–Loève (K‐L) decomposition, AS, and KAS, for the outputs defined in Equations (31)–(33)

Case	Mode 1	Mode 2	Mode 3
K‐L	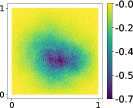	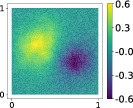	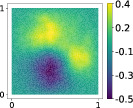
AS (31)	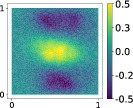	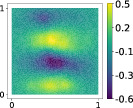	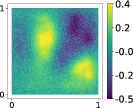
KAS (31)	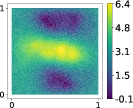	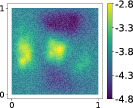	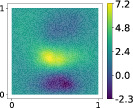
AS (32)	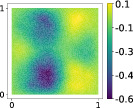	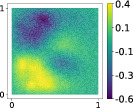	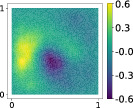
KAS (32)	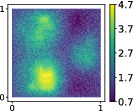	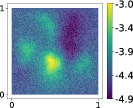	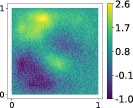
AS (33)	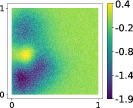	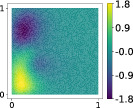	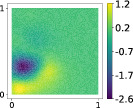
KAS (33)	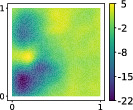	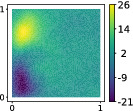	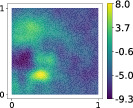

The third output is the evaluation of the solution at a specific degree of freedom with index î, that is

(33) $$f:{\mathbb{R}}^{m}\to \mathbb{R},\;f(X)=u_{î}\in \mathbb{R},$$

in this case the dimension of the input space is m=100. Since we use a Lagrangian basis in the finite element formulation and the polynomial order is 1, the node of the mesh associated to the chosen degree of freedom has coordinates [0.27,0.427]∈Ω. Qualitatively we can see from Table 3 that the AS modes locate features in the domain which are relatively more regular with respect to the KAS modes. To obtain this result, we increased the dimension of the input space, otherwise not even the AS modes could locate properly the position in the domain Ω of the degree of freedom.

In the second and third case the diffusion coefficient is given by

(34) $$\kappa (x)=\exp\left(\overset{D}{\underset{i=1}{\sum }}v_{j}[i]{\tilde{\psi }}_{j}(x)\right),\;\forall (s,x)\in \Omega \times \Omega ,$$

where $v_{j}\in {\mathbb{R}}^{D}$, j∈{1,…,D}, is the jth active eigenvector from the KAS procedure and the functions $\tilde{\Psi }:=({\tilde{\psi }}_{1},\ldots ,{\tilde{\psi }}_{D})$ are defined by

(35) $$\tilde{\Psi }=\phi (\Psi ),$$

where ϕ is the feature map defined in Equation (16) with the projection matrix W and bias b, and $\Psi :=(\gamma_{1}\psi_{1},\ldots ,\gamma_{m}\psi_{m})$.

The gradients of the three outputs of interest considered are evaluated with the adjoint method.

## 5 A CFD PARAMETRIC APPLICATION OF KAS SOLVED WITH THE DG METHOD

We want to test the kernel‐based extension of the active subspaces in a CFD context. The lift and drag coefficients of a NACA 0012 airfoil are considered as model functions. Numerical simulations are carried out with different input parameters for quantities that describe the geometry and the physical conditions of the problem. The evolution of the model is protracted until a periodic regime is reached. Once the simulation data have been collected, sensitivity analysis is performed searching for an active subspace and response surfaces with GPR are then built from the application of AS and KAS techniques.

The fluid motion is modeled through the unsteady incompressible Navier–Stokes equations approximated through the Chorin–Temam operator‐splitting method implemented in HopeFOAM.^67^ HopeFOAM is an extension of OpenFOAM,^68^, ^69^ an open source software for the solution of complex fluid flows problems, to variable higher order element method and it adopts a DG method, based on the formulation proposed by Hesthaven and Warburton.^52^

The DG method is a high‐order method, which has appealing features such as the low artificial viscosity and a convergence rate which is optimal also on unstructured grids, commonly used in industrial frameworks. In addition to this, DG is naturally suited for the solution of problems described by conservative governing equations (Navier–Stokes equations, Maxwell's equations, and so on) and for parallel computing. All these properties are due to the fact that, differently from formulations based on standard finite elements, no continuity is imposed on the cell boundaries and neighboring elements only exchange a common flux. The major drawback of DG is its high computational cost with respect to continuous Galerkin methods, due to the need of evaluating fluxes during each time step and the presence of extra degree of freedoms in correspondence of the elemental edges.

Nowadays, efforts are aimed at applying the DG in problems which involve deformable domains^70^ and at improving the computational efficiency of the DG adopting techniques based on hybridization methods, matrix‐free implementations, and massive parallelization.^71^, ^72^

### 5.1 Domain and mesh description

The domain Ω of the fluid dynamic simulation is a two‐dimensional duct with a sudden area expansion and a NACA 0012 airfoil is placed in the largest section. The inflow $\partial \Omega_{I}$ is placed at the beginning of the narrowest part of the duct, and here the fluid velocity is set constant along all the inlet boundary. The outlet is placed on the right‐hand side and it is denoted with $\partial \Omega_{O}$. We refer with $\partial \Omega_{W}:=\partial \Omega \setminus \{\partial \Omega_{O}\cup \partial \Omega_{I}\}$ to the boundaries of the airfoil and to the walls of the duct, where no slip boundary conditions are applied. The horizontal lengths of the sections of the channels are 0.6 and 1.35 m, respectively. The vertical length of the duct after the area expansion is 0.4 m, while the width of the first one depends on two distinct parameters. The airfoil has a chord‐length equal to 0.1 m but its position with respect to the duct and its angle of attack are described by geometric parameters. Further details about the geometric parameterization of the geometry are provided in the following section. A proper triangulation is designed with the aid of the gmsh^73^ tool and the domain is discretized with 4445 unstructured elements.

The evaluation of adimensional magnitudes, commonly used for characterizing the fluid flow field, requires the definition of some reference magnitudes. For the problem at hand, we consider the equivalent diameter of the channel in correspondence of the inlet as the reference lengthscale, while the reference velocity is the one imposed at the inlet.

### 5.2 Parameter space description

**FIGURE 7:**
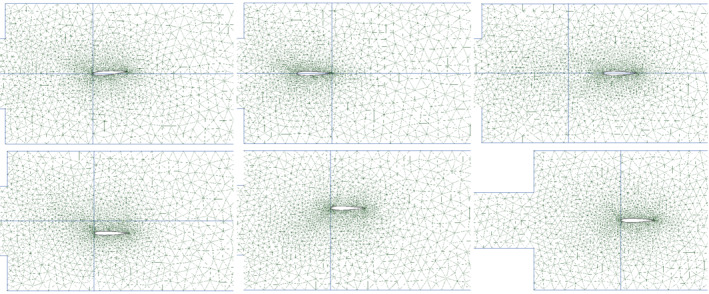
Domain configuration for minimum and maximum values of some geometric parameters. The maximum angle of attack α, the ranges for the horizontal translation $x_{0}$, the ranges for the vertical translation $y_{0}$, and the minimum opening of the channel which depends on the parameters $y^{+}$ and $y^{-}$ are represented in Table 4.

We chose seven heterogeneous parameters for the model: two physical, and five geometrical which describe the width of the channel and the position of the airfoil. In Table 4, the ranges for the geometrical and physical parameters of the simulation are reported. U is the first component of the initial velocity, ν is the kinematic viscosity, $x_{0}$ and $y_{0}$ are the horizontal and vertical components of the translation of the airfoil with respect to its reference position (see Figure 7), α is the angle of the counterclockwise rotation and the center of rotation is located right in the middle of the airfoil, $y^{+}$ and $y^{-}$ are the module of the vertical displacements of the upper and lower side of the initial conduct from a prescribed position.

**TABLE 4:** Parameter ranges for the NACA problem

	ν	U	$x_{0}$	$y_{0}$	α	$y^{+}$	$y^{-}$
Lower bound	0.00036	0.5	−0.099	−0.035	0	−0.02	−0.02
Upper bound	0.00060	2	0.099	0.035	0.0698	0.02	0.02

In Figure 7, the different configurations of the domain for the minimum and maximum values of the parameters α, $x_{0}$, $y_{0}$, and the minimum opening of the channel are reported.

We have considered only the counterclockwise rotation of the airfoil for symmetrical reasons. The range of the Reynolds number varies from 400 to 2000, still under the regime of laminar flow.

### 5.3 Governing equations

The CFD problem is modeled through the incompressible Navier–Stokes and the open source solver HopeFOAM^67^ has been employed for solving this set of equations.^52^

Let $\Omega \subset {\mathbb{R}}^{2}$ be the two‐dimensional domain introduced in Section 5.1, and let us consider the incompressible Navier–Stokes equations. Omitting the dependence on $(x,t)\in \Omega \times {\mathbb{R}}^{+}$ in the first two equations for the sake of compactness, the governing equations are

(36) $$\begin{array}{ll} \left\{\begin{array}{ll} \partial_{t}u+(u\cdot \nabla )u=-\nabla p+\nu \Delta u,\; & x\in \Omega ,\\ \nabla \cdot u=0,\; & x\in \Omega ,\\ u(x,0)=u_{0},\;p(x,0)=0,\; & x\in \Omega ,\\ u(x,t)=u_{0},\;n\cdot \nabla p(x,t)=0,\; & x\in \partial \Omega_{I},\\ u(x,t)=0,\;n\cdot \nabla p(x,t)=0,\; & x\in \partial \Omega_{W},\\ n\cdot \nabla u(x,t)=0,\;p(x,t)=1,\; & x\in \partial \Omega_{O}, \end{array}\right. & \end{array}$$

where p is the scalar pressure field, u=(u,v) is the velocity field, ν is the viscosity constant and $u_{0}$ is the initial velocity. In conservative form, the previous equations can be rewritten as

(37) $$\left\{\begin{array}{c} \partial_{t}u+\nabla \cdot ℱ=-\nabla p+\nu \Delta u,\\ \nabla \cdot u=0, \end{array}\right.$$

with the flux ℱ given by

(38) $$ℱ=\left[F_{1},F_{2}\right]=\left[\begin{array}{cc} u^{2} & uv\\ uv & v^{2} \end{array}\right].$$

From now on, in order to have a more compact notation, the advection term is written as 𝒩(u)=∇·ℱ(u).

For each timestep, the procedure is broken into three stages accordingly to the algorithm proposed by Chorin and adapted for a DG framework by Hesthaven and Warburton:^52^ the solution of the advection dominated conservation law component, the pressure correction weak divergence‐free velocity projection, and the viscosity update. The nonlinear advection term is treated explicitly in time through a second order Adams–Bashforth method,^74^ while the diffusion term implicitly. The Chorin algorithm is reported in Algorithm 6.

In order to recover the DG formulation, the equations introduced by the Chorin method are projected onto the solution space by introducing a proper set of test functions and then the variables are approximated over each element as a linear combination of local shape functions. The DG does not impose the continuity of the solution between neighboring elements and therefore it requires the adoption of methods for the evaluation of the flux exchange between neighboring elements. In the present work, the convective fluxes are treated accordingly to the Lax–Friedrichs scheme, while the viscous ones are solved through the interior penalty method.^75^, ^76^

Algorithm 6 **Chorin algorithm**

1

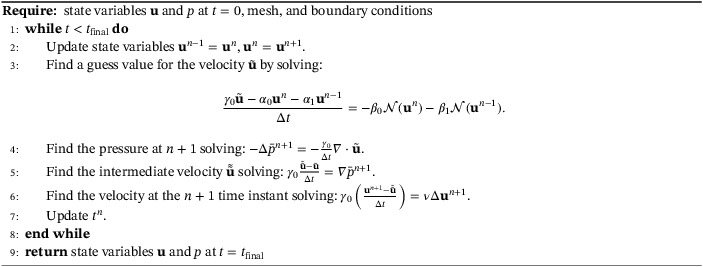

The aerodynamic quantities we are interested in are the lift and drag coefficients in the incompressible case computed from the quantities u, p, ν, $A_{\text{ref}}$, and $u_{0}$ with a contour integral along the airfoil Γ as

(39) $$f=\oint_{\Gamma }pn-\nu \left(\nabla u+\nabla u^{T}\right)n\;ds.$$

The vector n is the outward normal along the airfoil surface. The circulation in Γ is affected by both the pressure and stress distributions around the airfoil. The projection of the force along the horizontal and vertical directions gives the drag and lift coefficients, respectively

(40) $$C_{D}=\frac{f\cdot e_{1}}{\frac{1}{2}|u_{0}{|}^{2}A_{\text{ref}}},$$

(41) $$C_{L}=\frac{f\cdot e_{2}}{\frac{1}{2}|u_{0}{|}^{2}A_{\text{ref}}},$$

where the reference area $A_{\text{ref}}$ is the chord of the airfoil times a length of 1 m. For the aerodynamic analysis of the fluid flow past an airfoil, see Reference 77.

### 5.4 Numerical results

In this section, a brief review of the procedure and some details about the numerical method and the computational domain will be presented along the results obtained. For what concerns the DG the polynomial order chosen is 3. The total number of degrees of freedom is 133,350. Small variations on the mesh are present in each of the 285 simulations due to the different configurations of the domain. Each simulation is carried out until a periodic behavior is reached and for this reason the final times range between 3.5 and 5 s, depending on the specific configuration. The integration time intervals are variable and they are updated at the end of each step in order to satisfy the CFL condition. The seven physical and geometrical parameters of the simulation are sampled uniformly from the intervals in Table 4. In total, we consider a dataset of 285 samples.

**FIGURE 8:**
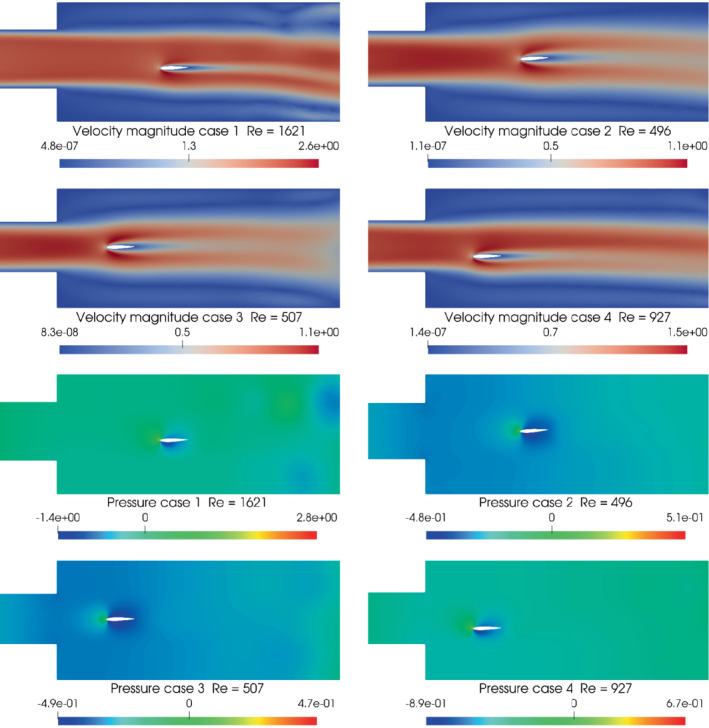
Module of the velocity fields (on the left) and pressure fields (on the right) evaluated at the last time instant of four different simulations. The corresponding parameters are reported in Table 5.

With the purpose of qualitatively visualizing the results, four different simulations are reported in Figure 8 for the module of the velocity field and the scalar pressure field, respectively, both evaluated at the last time instant. These simulations were chosen from the 285 collected in order to show significant differences in the evolution of the fluid flow. In Table 5, the corresponding parameters are reported. Depending on the position of the airfoil and the other physical parameters, different fluid flow patterns can be qualitatively observed.

**TABLE 5:** Parameters associated to the simulations plotted in Figure 8

#	ν	U	$x_{0}$	$y_{0}$	α	$y^{+}$	$y^{-}$
1	0.000405	1.99	−0.096	−0.00207	0.00282	0.00784	0.0188
2	0.000541	0.763	−0.084	0.00279	0.0260	−0.0108	0.0195
3	0.000406	0.533	−0.0503	−0.0327	0.0604	−0.0193	0.0068
4	0.000430	1.11	−0.0897	−0.0279	0.0278	−0.00624	0.0197

The lift ($C_{L}$) and drag ($C_{D}$) coefficients are evaluated when stationary or periodic regimes are reached, starting from the values of pressure and viscous stresses evaluated on the nodes close to the airfoil. After this sensitivity analysis is carried out. First the AS method is applied. The gradients necessary for the application of the AS method are obtained from the Gaussian process regression of the model functions $C_{L}$ and $C_{D}$ on the whole parameters' domain. The eigenvalues of the uncentered covariance matrix for the lift and drag coefficients suggest the presence of a one‐dimensional active subspace in both cases.

**FIGURE 9:**
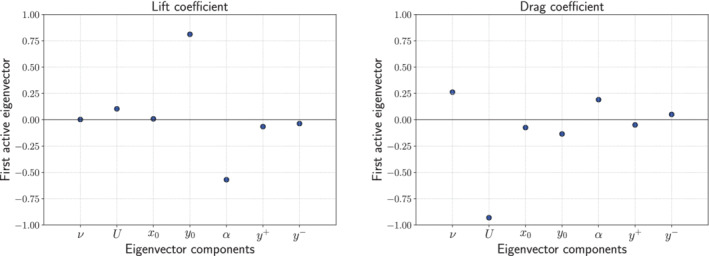
Components of the first active eigenvector for the lift coefficient (on the left), and for the drag coefficient (on the right). Values near 0 suggest little sensitivity for the target function.

The plots of the first active eigenvector components are useful as sensitivity measures, see Figure 9. The greater the absolute value of a component is, the greater is its influence on the model function. We observe that the lift coefficient is influenced mainly by the vertical position of the airfoil and the angle of attack, while the drag coefficient depends mainly on the initial velocity, and secondarily on the viscosity and on the angle of attack.

As one could expect from physical considerations, the angle of attack affects both drag and lift coefficients, while the viscosity, which governs the wall stresses, is relevant for the evaluation of the $C_{D}$. The vertical position of the airfoil with respect to the symmetric axis of the section of the duct after the area expansion also greatly affects both coefficients, and this is mainly due to the fact that the fluid flow conditions change drastically between the core, where the speed is higher, and the one close to the wall of the duct, where the speed tends to zero. On the other hand, the horizontal translation has almost no impact on the results, given the regularity of the fluid flow along the x‐axis for the considered range of $x_{0}$. Moreover, the nonsymmetric behavior of the upper and lower parameters which determine the opening of the channel is due to the nonsymmetric choice of the range considered for the angle of attack.

The KAS method was applied with 1500 features. In order to compare the AS and KAS methods 5‐fold cross validation was implemented. The score of cross validation is the RRMSE defined in Equation (19).

**FIGURE 10:**
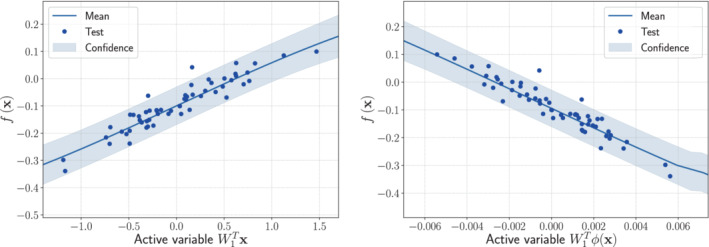
Comparison between the sufficiency summary plots obtained from the application of AS and KAS methods for the lift coefficient $C_{L}$ defined in Equation (41). The left plot refers to AS, the right plot to KAS. With the blue solid line, we depict the posterior mean of the GP, with the shadow area the c68% confidence intervals, and with the blue dots the testing points.

**FIGURE 11:**
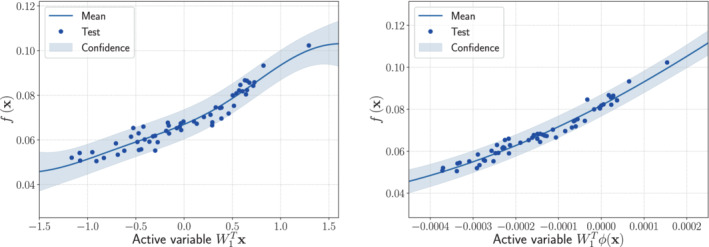
Comparison between the sufficiency summary plots obtained from the application of AS and KAS methods for the drag coefficient $C_{D}$ defined in Equation (40). The left plot refers to AS, the right plot to KAS. With the blue solid line, we depict the posterior mean of the GP, with the shadow area the c68% confidence intervals, and with the blue dots the testing points.

The GPR for the two methods are shown in Figure 10 for the lift coefficient, and in Figure 11 for the drag coefficient. They were obtained as a single step of 5‐fold cross validation with one fifth of the 285 samples used as test set. The spectral distribution of the feature map is the Gaussian distribution for the lift, and the Beta for the drag, respectively. The RRMSE mean and standard deviation from 5‐fold cross validation, are reported for different active dimensions in Table 6. The feature map from Equation (16) was adopted. The hyperparameters of the spectral distributions were tuned with logarithmic grid‐search with 5‐fold cross validation as described in Algorithm 5.

**TABLE 6:** Summary of the results for AS and KAS procedures

Method	Dim	Feature space dim	Lift spectral distribution	RRMSE lift	Drag spectral distribution	RRMSE drag
AS	1	–	–	0.37 ± 0.09	–	0.268 ± 0.032
KAS	1	1500	$𝒩(0,\lambda I_{d})$	**0.344** ± 0.048	Beta(α,β)	**0.218** ± 0.045
AS	2	–	–	0.384 ± 0.073	–	0.183 ± 0.027
KAS	2	1500	$𝒩(0,\lambda I_{d})$	**0.328** ± 0.071	Beta(α,β)	**0.17** ± 0.02

*Note*: The best results are given in bold.

Regarding the drag coefficient, the relative gain using the KAS method reaches the 19.2% on average when employing the Beta spectral measure for the definition of the feature map. The relative gain of the one dimensional response surface built with GPR from the KAS method is 7% on average for the lift coefficient. This result could be due to the higher noise in the evaluation of the $C_{L}$. In this case, the relative gain increases when the dimension of the response surface increases to 2 with a gain of 14.6%. A slight reduction of the AS RRMSE relative to the drag coefficient is ascertained when increasing the dimension of the response surface.

## 6 CONCLUSIONS AND PERSPECTIVES

In this work, we presented a new nonlinear extension of the active subspaces property that introduces KAS. The method exploits random Fourier features to find active subspaces on high‐dimensional feature spaces. We tested the new method over five different benchmarks of increasing complexity, and we provided pseudo‐codes for every aspects of the proposed kernel‐extension. The tested model functions range from scalar to vector‐valued. We also provide a CFD application discretized by the DG method. We compared the kernel‐based active subspaces to the standard linear active subspaces and we observed in all the cases an increment of the accuracy of the Gaussian response surfaces built over the reduced parameter spaces. The most interesting results regard the possibility to apply the KAS method when an active subspace does not exist. This was shown for radial symmetric model functions.

Future developments will involve the study of more efficient procedures for tuning the hyperparameters of the spectral distribution. Other possible advances could be done finding an effective back‐mapping from the targets to the actual parameters in the full original space. This could promote the implementation of optimization algorithms or other parameter studies enhanced by the kernel‐based active subspaces extension.

## CONFLICT OF INTEREST

The authors declare no potential conflict of interest.

## Data Availability

The data that support the findings of this study are available from the corresponding author upon reasonable request.

## ENDNOTES

* Some authors refer to the active subspaces method, we prefer to employ the term active subspaces property, as suggested by Constantine.

† In this work with ℳ(m×n), we denote the set of real matrices with m rows and n columns.

‡ The dataset was taken from https://github.com/paulcon/as‐data‐sets.

## PROOF DETAILS

### 1

In this section, we provide an expanded version of the proof of Theorem 1.

The proof is remodeled from References 11 and 53, and it is developed in five steps:

1. Since $R_{V}\in ℳ(d,d)$ is symmetric positive definite there exists a basis of eigenvectors ${\left(w_{i}\right)}_{i\in \{1,\ldots ,d\}}$ and a corresponding set of positive eigenvalues ${\left(\beta_{i}\right)}_{i\in \{1,\ldots ,d\}}$ such that
  (A1)
  $$R_{V}=\overset{d}{\underset{i=1}{\sum }}\beta_{i}\;w_{i}⊗w_{i}.$$
2. Let us define the ridge approximation error as
  (A2)
  $$\begin{array}{ll} e=\|f-h\circ P_{r}{\|}_{L^{2}({\mathbb{R}}^{m},ℬ({\mathbb{R}}^{m}),\rho ;V)}={𝔼}_{P}\left[\|(f(X)-h(P_{r}(X)){\|}_{R_{V}}^{2}\right]. & \end{array}$$
  Then we can decompose the error analysis for each component employing the spectral decomposition (A1)
  (A3)
  $$\begin{array}{ll} {𝔼}_{P}\left[\|e(X){\|}_{R_{V}}^{2}\right] & ={𝔼}_{P}\left[\text{tr}(\left(R_{V}e(X)\right)⊗e(X))\right]\\ & =\overset{d}{\underset{i=1}{\sum }}\beta_{i}\;{𝔼}_{P}\left[\text{tr}(((w_{i}⊗w_{i})e(X))⊗e(X))\right]\\ & =\overset{d}{\underset{i=1}{\sum }}\beta_{i}\;{𝔼}_{P}\left[\left(w_{i}\cdot e(X))\;\text{tr}(w_{i}⊗e(X)\right)\right]\\ & =\overset{d}{\underset{i=1}{\sum }}\beta_{i}\;{𝔼}_{P}\left[{\left(w_{i}\cdot e(X)\right)}^{2}\right], \end{array}$$
  so we can define $e_{i}(X)=w_{i}\cdot e(X)=f_{i}(X)-h_{i}(P_{r}(X)),\;\forall i\in \{1,\ldots ,d\}$ and treat each component separately.
3. The next step involves the application of Lemma 1 to the scalar functions $f_{i}(X)-h_{i}(P_{r}(X)),\;\forall i\in \{1,\ldots ,d\}$
  (A4)
  $$\begin{array}{ll} {𝔼}_{P}\left[{\left(f_{i}(X)-h_{i}(P_{r}(X))\right)}^{2}\right] & ={𝔼}_{P}\left[{𝔼}_{P}\left[{\left(f_{i}(X)-h_{i}(P_{r}(X))\right)}^{2}|\sigma (P_{r})\right]\right]\\ & \leq {𝔼}_{P}\left[C_{p}(\rho ,P_{r}(X)){𝔼}_{P}\left[\left\|(I-P_{r}^{T})\nabla f_{i}(X){\|}_{2}^{2}|\sigma (P_{r}\right)\right]\right]\\ & \leq {𝔼}_{P}{\left[C_{p}(\rho ,P_{r}(X))\right]}^{\frac{1}{p}}\;{𝔼}_{P}{\left[\|(I-P_{r}^{T})\nabla f_{i}(X){\|}_{2}^{2}\right]}^{\frac{1}{q}}, \end{array}$$
  where we used the Hölder inequality with indexes (p,q)=(∞,1) when ρ belongs to the first and second classes of Assumption 3, and $(p,q)=\left(\frac{\tau +1}{\tau },1+\tau \right)$ when ρ belongs to the third class.
  Then we can bound ${𝔼}_{P}{\left[\left(C_{p},P_{r}(X)\right)\right]}^{\frac{1}{p}}$ with a constant $C(C_{p}(\rho ,P_{r}(X)))$ which depends on the class of ρ (see Lemmas 3.1, 4.2–4.4 and Theorem 4.5 of Reference 53) as follows
  (A5)
  $$\begin{array}{ll} & {𝔼}_{P}{\left[C_{p}(\rho ,P_{r}(X))\right]}^{\frac{1}{p}}\;{𝔼}_{P}{\left[\|(I-P_{r}^{T})\nabla f_{i}(X){\|}_{2}^{2}\right]}^{\frac{1}{q}}\\ & \;\leq C(C_{p}(\rho ,P_{r}(X)))\;\text{tr}({𝔼}_{P}{\left[\left(I-P_{r}^{T})\nabla f_{i}(X){\left(\nabla f_{i}(X)\right)}^{T}(I-P_{r}\right)\right]}^{\frac{1}{q}})\\ & \;=C(C_{p}(\rho ,P_{r}(X)))\;\text{tr}{\left((I-P_{r}^{T}){𝔼}_{P}\left[\nabla f_{i}(X){\left(\nabla f_{i}(X)\right)}^{T}\right](I-P_{r})\right)}^{\frac{1}{q}}. \end{array}$$
4. The spectral decomposition (A1) is employed again and the covariance matrix H is introduced in the last equation
  (A6)
  $$\begin{array}{ll} & {𝔼}_{P}\left[\|e(X){\|}_{R_{V}}^{2}\right]\\ & \;\leq \overset{d}{\underset{i=1}{\sum }}\beta_{i}\;C(C_{p}(\rho ,P_{r}(X)))\;\text{tr}{\left((I-P_{r}^{T}){𝔼}_{P}\left[\left({\left(\nabla f(X)\right)}^{T}w_{i})⊗({\left(\nabla f(X)\right)}^{T}w_{i}\right)\right](I-P_{r})\right)}^{\frac{1}{q}}\\ & \;=C(C_{p}(\rho ,P_{r}(X)))\;\text{tr}{\left((I-P_{r}^{T}){𝔼}_{P}\left[{\left(\nabla f(X)\right)}^{T}\left(\overset{d}{\underset{i=1}{\sum }}\beta_{i}^{q}\;w_{i}⊗w_{i}\right)\nabla f(X)\right](I-P_{r})\right)}^{\frac{1}{q}}\\ & \;=C(C_{p}(\rho ,P_{r}(X)))\;\text{tr}{\left((I-P_{r}^{T}){𝔼}_{P}\left[{\left(\nabla f(X)\right)}^{T}R_{V}(\rho )\nabla f(X)\right](I-P_{r})\right)}^{\frac{1}{q}}\\ & \;=C(C_{p}(\rho ,P_{r}(X)))\;\text{tr}{\left((I-P_{r}^{T})H(I-P_{r})\right)}^{\frac{1}{q}}, \end{array}$$
  where $R_{V}(\rho )$ is the original metric matrix if ρ belongs to the first or second class of Assumption 3 and is equal to
  (A7)
  $$\overset{d}{\underset{i=1}{\sum }}\beta_{i}^{1+\tau }\;w_{i}⊗w_{i},$$
  if ρ belongs to the third class.
5. Finally the bound in the statement of the theorem is recovered solving the following minimization problem with classical model reduction arguments employing SVD
  (A8)
  $${\tilde{P}}_{r}=\underset{P_{r}\in 𝒪(m,m)}{\text{arg min}}\text{tr}((I-P_{r}^{T})H(I-P_{r})).$$
